# DNA binding by *Corynebacterium glutamicum *TetR-type transcription regulator AmtR

**DOI:** 10.1186/1471-2199-10-73

**Published:** 2009-07-23

**Authors:** Daniela Muhl, Nadja Jeßberger, Kristin Hasselt, Christophe Jardin, Heinrich Sticht, Andreas Burkovski

**Affiliations:** 1 Lehrstuhl für Mikrobiologie, Friedrich-Alexander-Universität Erlangen-Nürnberg, Erlangen, Germany; 2 Institut für Biochemie, Friedrich-Alexander-Universität Erlangen-Nürnberg, Erlangen, Germany

## Abstract

**Background:**

The TetR family member AmtR is the central regulator of nitrogen starvation response in *Corynebacterium glutamicum*. While the AmtR regulon was physiologically characterized in great detail up to now, mechanistic questions of AmtR binding were not addressed. This study presents a characterization of functionally important amino acids in the DNA binding domain of AmtR and of crucial nucleotides in the AmtR recognition motif.

**Results:**

Site-directed mutagenesis, the characterization of corresponding mutant proteins by gel retardation assays and surface plasmon resonance and molecular modelling revealed several amino acids, which are directly involved in DNA binding, while others have more structural function. Furthermore, we could show that the spacing of the binding motif half sites is crucial for repression of transcription by AmtR.

**Conclusion:**

Although the DNA binding domain of TetR-type repressors is highly conserved and a core binding motif was identified for AmtR and TetR(D), the AmtR binding domain shows individual properties compared to other TetR proteins. Besides by distinct amino acids of AmtR, DNA binding is influenced by nucleotides not only of the conserved binding motif but also by spacing nucleotides in *C. glutamicum*.

## Background

Almost all of the macromolecules in a bacterial cell, e.g. proteins, nucleic acids and cell wall components, contain nitrogen. Thus, prokaryotes have developed elaborate mechanisms to provide an optimal nitrogen supply for metabolism and to overcome and survive situations of nitrogen limitation, generally summarized as nitrogen control. This communication focuses on nitrogen control in *Corynebacterium glutamicum*, a Gram-positive soil bacterium used for the industrial production of amino acids [1]. We have been studying nitrogen metabolism and nitrogen regulation in corynebacteria with a focus on *C. glutamicum *for several years (for recent reviews, see [2-4]) and could show that transcription of genes in response to nitrogen limitation is governed in corynebacteria, i.e. *C. glutamicum*, *Corynebacterium efficiens *and *Corynebacterium diphtheriae*, by TetR-type regulator AmtR [5,6], which blocks transcription of various genes during growth in nitrogen-rich medium. The AmtR regulon of *C. glutamicum *was characterized by a combination of bioinformatics and molecular biology approaches. At least 35 genes, which encode transporters and enzymes for ammonium assimilation (*amtA*, *amtB*, *glnA*, *gltBD*, *dapD*), creatinine (*codA*, *crnT*) and urea metabolism (*urtABCDE*, *ureABCEFGD*), a number of biochemically uncharacterized enzymes and transport systems as well as signal transduction proteins (*glnD*, *glnK*), are directly controlled by the AmtR protein in *C. glutamicum *[7,8].

An AmtR binding site consensus motif was deduced from bioinformatic analyses of available genome sequence information and competitive gel retardation assays [7,8]. The resulting AmtR box with the nucleotide sequence tttCTATN_6_AtAGat/aA (with bases represented by capital letters being highly conserved) is a more or less palindromic sequence and can be located in the promoter region either on the sense or antisense strand.

In this study we address the question which amino acids within the AmtR DNA binding domain are in fact contacting the DNA and why AmtR expression is not controlled by an autoregulatory circuit as found for other TetR-type regulators (for review, see [9])

## Results

### Characterization of the AmtR binding domain reveals functionally important amino acid residues

**Figure 1:**
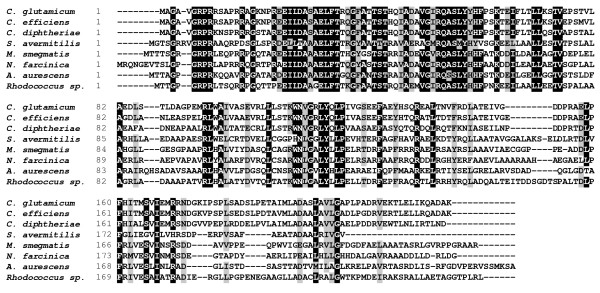
**Sequence alignment of AmtR proteins from different Gram-positive bacteria**. Amino acid residues identical in all sequences are shaded in black, other conserved amino acids in gray.

**Figure 2:**
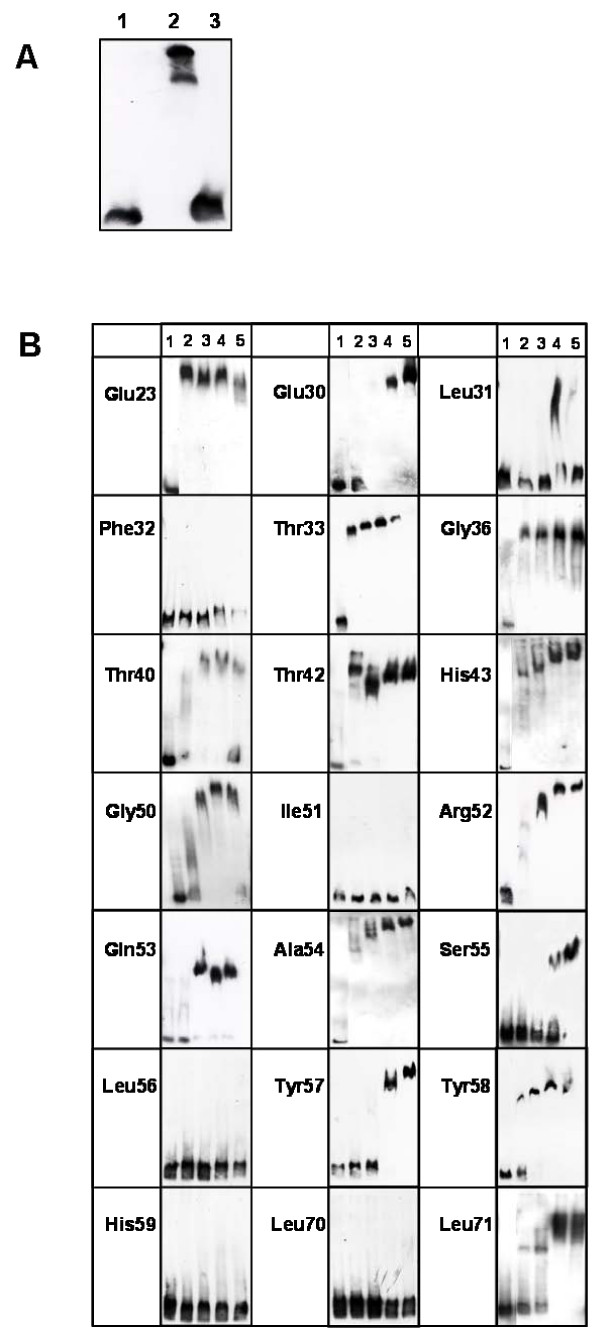
**Gelretardation assays using AmtR variants**. **(A) **A DNA fragment spanning nucleotides -298 to -1 relative to the start codon of *amtB *(0.04 ng DNA per lane) was used for the gel shift assay (1) negative control: 50 ng (2170 nM) of MBP, (2) 150 ng (2170 nM) wild type AmtR fused to MBP, (3) *amtB *upstream DNA without added protein. **(B) **Recombinant AmtR-MBP proteins carrying alanine exchanges of the indicated amino acid residues (with exception of Ala54, which was altered to glycine). DNA as described above plus (1) 0 ng, (2) 150 ng, (3) 750 ng, (4) 1950 ng, (5) 3 μg of the indicated AmtR*-MBP fusion.

As a molecular biology approach to identify amino acid residues of AmtR involved in DNA binding, site-directed mutagenesis experiments were carried out. Amino acids highly conserved in the DNA binding domain of TetR family proteins (Fig. 1) were selected and exchanged against alanine, with exception of the conserved Ala54 residue, which was changed to glycine. Wild type AmtR and AmtR variants were purified as maltose binding protein (MBP) fusions and applied in gel retardation experiments. As target sequence a PCR fragment spanning nucleotides -298 to -1 relative to the start codon of the *amtB *gene and comprising three AmtR binding sites [4,7] was used. While 150 ng of wild type AmtR-MBP led to a complete retardation of 0.04 ng of target DNA, the same amount of MBP had no effect (Fig. 2A). Subsequently carried out gel retardation experiments with rising amounts of AmtR variants (addition of AmtR*-MBP up to 3 μg) revealed that exchange of residues Glu23, Thr33, Gly36 and Thr42 had no effect. The corresponding recombinant proteins behaved as wild type. A slightly reduced affinity compared to wild type AmtR was observed for AmtR* with exchange of His43 and Arg52 to alanine and Ala54 to glycine, while significantly reduced binding was observed for alterations of Glu30, Leu31, Thr40, Gly50, Gln53, Ser55, Tyr57, Tyr58 and Leu71. Mutations resulting in an alanine exchange at positions Phe32, Ile51, Leu56, His59 and Leu70 led to a complete loss of AmtR* binding (Fig. 2B).

From these gel retardation assays, K_D _values for the binding of AmtR-MBP and selected AmtR*-MBP variants were estimated. The equilibrium binding constant for AmtR-MBP was 2.4 × 10^-6 ^M and 2.2 × 10^-6 ^M for the variant carrying the Thr33Ala exchange, which is in accordance with the wild type-like behaviour of this protein in the gel retardation assay. With rising effect on binding, increasing K_D _values were estimated, e. g. 1.0 and 2.0 × 10^-5 ^M for exchange Glu30 and Leu71, as well as 7.1 × 10^-5 ^M for AmtR*-MBP carrying the Ser55Ala exchange.

**Figure 3:**
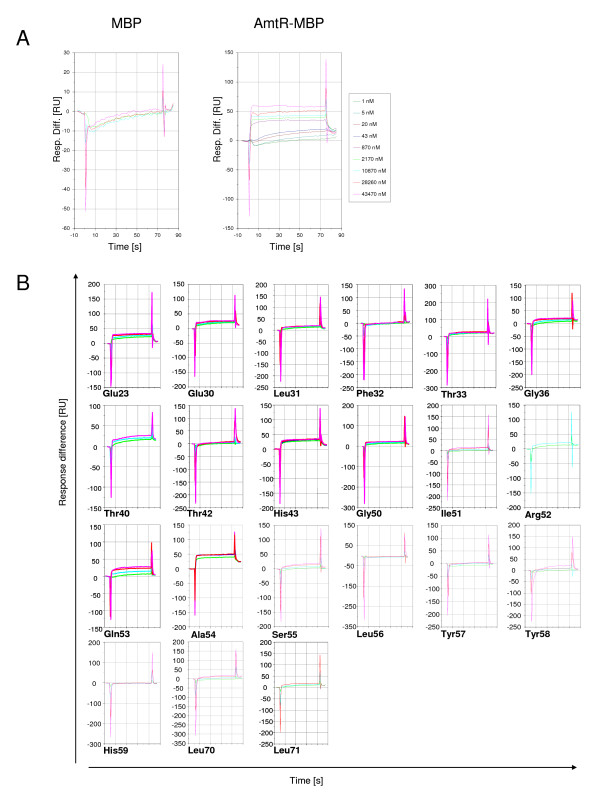
**Surface plasmon resonance measurements**. An *amtB *promoter fragment spanning nucleotides -186 to -156 (relative to the start codon) was immobilized on Biacore chips rising concentrations of protein were added (for colour code, see Fig. 3A). **(A) **Binding properties of negative control (MBP) and positive control (AmtR-MBP fusion), **(B) **Influence of amino acid exchanges on binding of AmtR variants. Colour code for the concentrations of proteins added as in (A).

As an independent assay, surface plasmon resonance (SPR) measurements were carried out. In these experiments, the same AmtR-MBP preparations as in the gel retardation assays were used, while the DNA immobilized on the chip surface corresponded to a shorter fragment of the *amtB *promoter resembling only one AmtR binding site (nucleotide position -186 to -156, [4]). Again, purified maltose binding protein was used as negative control and AmtR-MBP as positive control (Fig. 3A). MBP did not bind to the immobilized DNA, while addition of AmtR-MBP resulted in a clearly dose-dependent increase in response units, indicating binding of the protein to its target DNA. Similarly, binding of AmtR variants was tested (Fig. 3B). In general, results obtained with this approach were similar to that described above for the gel retardation assays. However, for exchange of Thr42, Ser55, Tyr57 and Tyr58 a stronger reduction of binding was observed, which might indicate a stabilization effect or cooperative binding of AmtR-MBP, when more than one binding site is available.

In summary, the analysis of AmtR variants generated by site-directed mutagenesis and analyzed by gel retardation experiments and SPR measurements hint to a crucial role of several amino acids in DNA binding. However, it was difficult to differ between direct and indirect effects. Therefore, modelling experiments were carried out.

### Molecular modelling indicates the function of distinct amino acids

**Figure 4:**
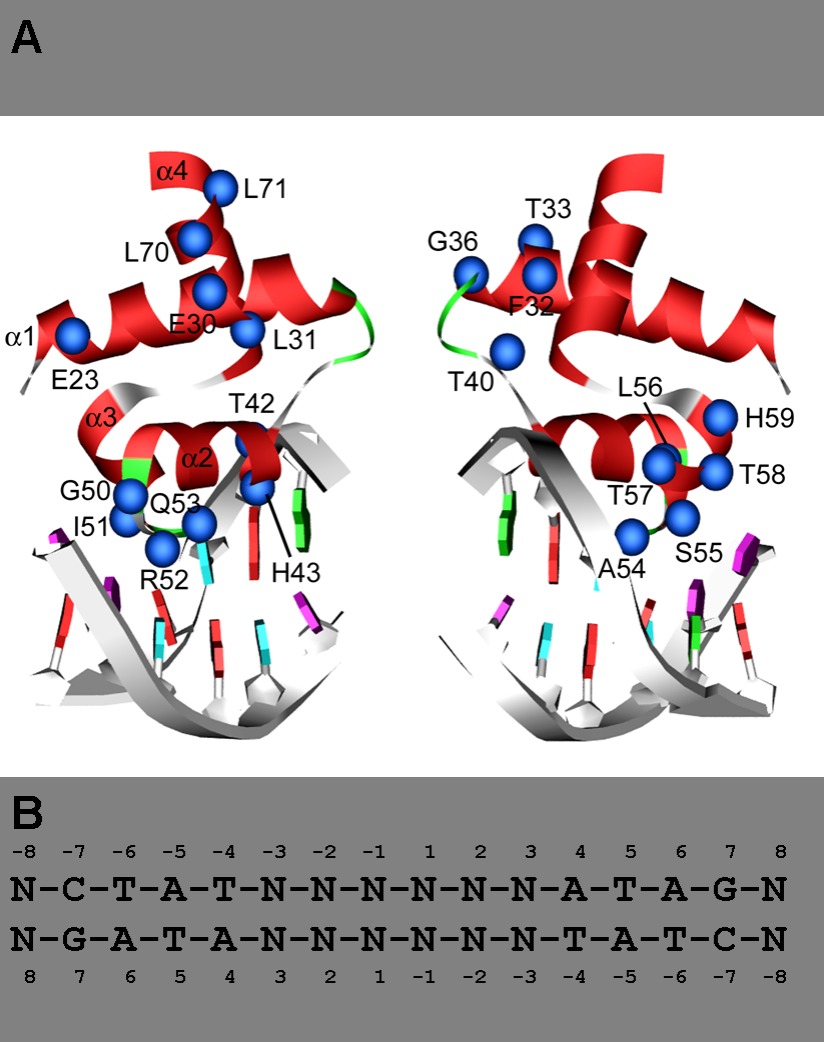
**Homology model of the AmtR repressor-operator complex**. **(A) **Three-dimensional model of AmtR DNA binding domain in complex with DNA. The two binding sites of dimeric AmtR are shown separately on the left and right half of the picture. The protein is depicted in backbone presentation and sequence positions that were experimentally investigated are shown as balls. The DNA backbone is shown as grey ribbon and the bases are colored according to their type. **(B) **Sequence of the AmtR operator indicating the numbering scheme used in the present work. The bases of the conserved "CTAT" recognition motif are explicitly labeled, while the remaining nonconserved bases are denoted as "N".

In order to allow a structural interpretation of the AmtR mutation data, we started a molecular modeling approach of AmtR in complex with DNA based on the crystal structure of the TetR-DNA complex. The significant sequence identity between the DNA-binding domains of the proteins and the fact that they recognize an identical "CTAT" core motif allowed the calculation of a molecular model (Fig. 4), which provides the basis for subsequent detailed analysis.

The model shares the same characteristic structural properties previously reported for the TetR-DNA crystal structure: The DNA-binding domains are constituted of helices α1 to α4 of the N-terminal domains. The binding motif itself consists of the α2-α3 loops (amino acid residues Thr42 to His59) organized in helix-turn-helix (HTH) motifs whereas helix 4 constitutes the link between the N- and C-terminal domains. Each HTH motif of the repressor binds to one major groove of the palindromic DNA consensus nucleotide sequence "CTAT". Due to this twofold symmetry, subsequent bioinformatic analysis was restricted to one half-site of the complex.

**Figure 5:**
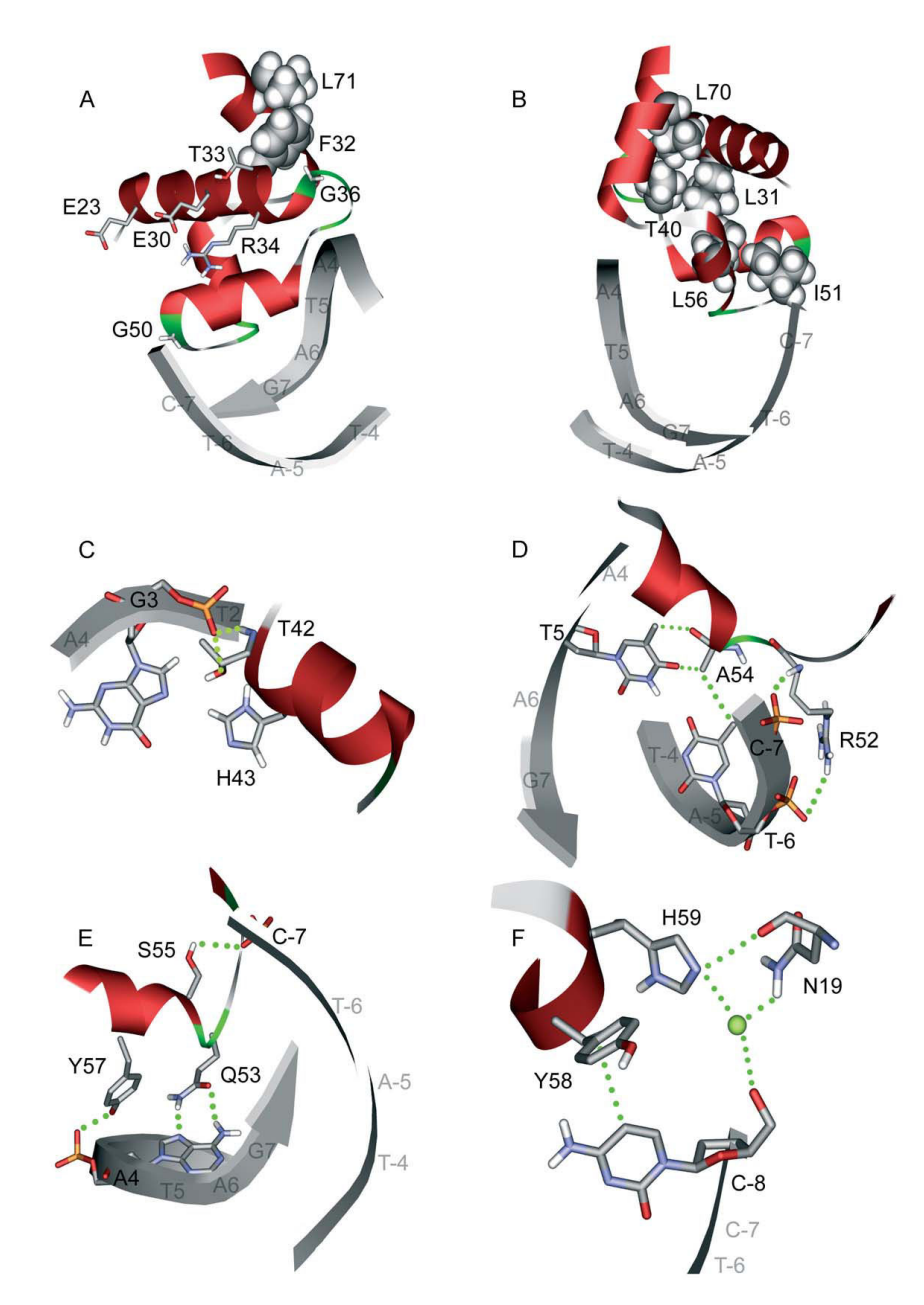
**Detailed structural analysis of different residues in the DNA binding domain of AmtR**. Panels **(A) **and **(B)**: Location and interactions of the non-interface residues that were experimentally investigated. Surface-exposed residues are depicted as sticks, while those residues that are buried in the interior of the protein are shown in space-filled presentation. Panels **(C) **to **(F)**: Interactions of the residues located in the protein DNA interface. The DNA backbone is shown as grey tube and nucleotides of the binding site are shown in stick presentation. Contacts are indicated by dotted lines. The residues shown in **(F) **might play a dual role by forming both contacts with the DNA and with other parts of the protein. A water molecule, which was modeled in analogy to the TetR-operator complex crystal structure, is shown as green ball. See text for more details.

The AmtR-DNA model complex reveals that 9 of the 21 residues investigated are in contact with the DNA while the remaining 12 residues are located within the HTH binding motif but do not directly bind DNA (Fig. 4; Table 1). These two groups were termed interface (IF) and non-interface (NI) residues and their functions are discussed separately below. For several non-interface residues (Glu23, Thr33, Gly36), replacement by alanine had no effect on DNA binding properties. Structural analysis reveals that these residues are solvent exposed and do not form crucial interactions within AmtR (Fig. 5a).

**Table 1:** Structural features and role for DNA binding of different AmtR residues.

**Sequence position**	**Effect on DNA binding**	**Location**	**Structural features**	**Equivalent residue in *E. coli *TetR**
Glu23	o	NI	solvent exposed, only few interactions	Ser8

Glu30	++	NI	Glu30-Arg34 salt bridge	Glu15

Leu31	++	NI	buried	Leu16

Phe32	+++	NI	buried	Leu17

Thr33	o	NI	solvent exposed, only few interactions	Asn18

Gly36	o	NI	C-terminus of helix α1; only few interactions	Gly21

Thr40	++	NI	buried	Leu25

Thr42	o(+)	IF	backbone interactions with *G3 *phosphate group	Thr27

His43	+	IF	*G3 *ring	Arg28

Gly50	++	NI	tight turn	Gly35

Ile51	+++	NI	buried	Ile36

Arg52	+	IF	backbone contact with *T-6 *phosphate group	Glu37

Gln53	++	IF	side chain contacts with *A4 *ring	Gln38

Ala54	+	IF	weak non-polar interactions with the methyl group of *T-6*	Pro39

Ser55	++(+)	IF	side chain contacts with *C-7*	Thr40

Leu56	+++	NI	buried	Leu41

Tyr57	++(+)	IF	side chain contacts with *A4 *and *T5*	Tyr42

Tyr58	++(+)	IF	side chain interactions with DNA and with His59	Trp43

His59	+++	IF	side chain hydrogen bonds to protein and DNA	His44

Leu70	+++	NI	buried	Leu55

Leu71	++	NI	partially buried	Ala56

The first and second column list the residues investigated and their experimentally determined effect on DNA binding. "o" indicates no effect, while increasing numbers of "+ signs qualitatively reflect the magnitude, by which DNA binding is decreased. Signs in parenthesis indicate minor differences between the two experimental methods used. The third and fourth column list the location of the residue in the structure as deduced from the AmtR-DNA model as well as key structural features that might be of relevance for DNA binding. "IF" and "NI" denote protein-DNA interface and non-interface residues, respectively. The last column shows the structurally equivalent residues in *E. coli *TetR.

A completely different situation is present for those non-interface residues, for which replacement by alanine leads to a complete loss of DNA binding (Phe32, Ile51, Leu56, Leu70). These residues are buried within the interior of the protein and form interactions that stabilize the tertiary structure of the HTH motif (Table 1; Fig. 5A, B). The respective interactions cannot be formed by alanine in the mutant protein structures, which will lead to a disruption of the three-dimensional structure of the binding domains consequently resulting in a (complete) loss of DNA binding activity.

For the remaining group of non-interface residues (Glu30, Leu31, Thr40, Gly50, Leu71), mutation results in weaker DNA binding. These residues are either buried in the protein structure (Leu31, Thr40, Leu71), or involved in the formation of a tight turn (Gly50) or a salt-bridge (Glu30). A replacement by alanine is therefore expected to have at least a moderate destabilizing effect for all of these sequence positions. The mechanism, by which this destabilization will reduce DNA binding affinity, cannot unambiguously be determined from the static model structure. One might speculate that mutation either leads to rearrangements within AmtR or that mutation might increase the portion of unfolded HTH domains, which are no longer capable of DNA binding. The observation that mutations can affect the disorder-order equilibrium of proteins has been recently also described for a mutation within the DNA binding domain of TetR as well [10].

The nine interface residues investigated can be divided into those, which have no or moderate effects on binding affinity (Thr42, His43, Arg52, Ala54) and those which have a strong effect (Gln53, Ser55, Tyr57, Tyr58, His59). The first group of residues generally forms only weak interactions with the DNA (Table 1; Fig. 5C, D), while Gln53, Ser55, and Tyr57 form tight contacts with the "CTAT"-motif (Table 1; Fig. 5E). Gln53 for example specifically recognizes the purine ring of *A4 *via two hydrogen bonds.

A special situation is observed for residues Tyr58 and His59, which form only weak contacts with the DNA, but nevertheless have a strong effect on binding affinity when mutated to alanine: Tyr58 forms only weak interactions with the ring of a non-conserved nucleotide at promoter position -8 that is located adjacent to the "CTAT"-motif (Fig. 5F). Tyr58, however, might play an additional role for stabilizing the HTH motif by interaction with His59. The side chain orientation of His59 appears to be particularly important, since this residue forms both a hydrogen bond with Asn19 at the N-terminus of helix α1, as well as a water-mediated interaction with a phosphoryl group (Fig. 5F) that was deduced in analogy to the TetR crystal structure. Mutation of His59 to alanine might therefore affect DNA binding both by direct and by indirect effects thereby explaining the strong influence of this mutation.

### The lack of AmtR autoregulation is caused by variations of spacing nucleotides rather than variation of the core binding motif

**Figure 6:**
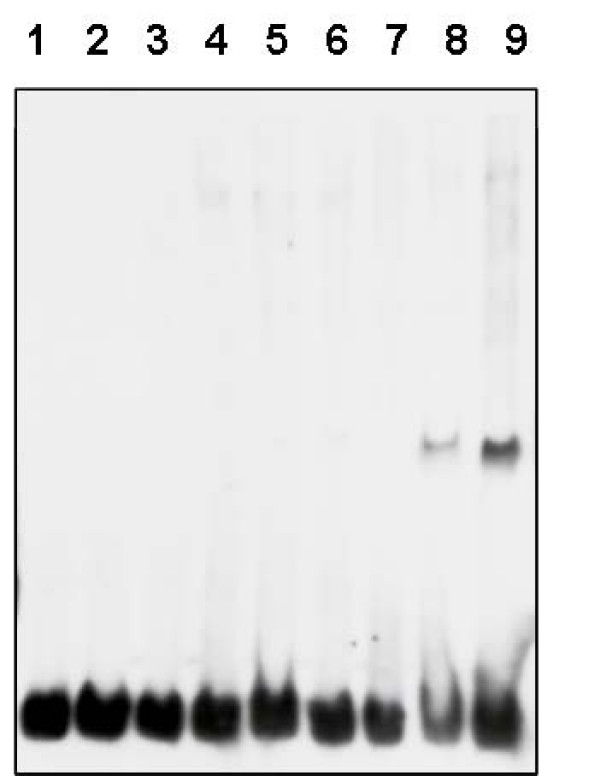
**Gelretardation assays using *amtR *upstream DNA variants**. (1–3) 0.04 ng of native DNA, (4–6) *amtR *upstream DNA carrying a G to T exchange at position 2, (7–9) *amtR *upstream DNA with additional spacing nucleotide. (1, 4, 7) without AmtR-MBP, (2, 5, 8) with 150, (3, 6, 9) with 300 ng of AmtR-MBP.

Compared to other TetR family members, *C. glutamicum *AmtR lacks an autoregulation circuit (for review, see [9]). Neither with DNA microarrays nor with real-time reverse transcriptase PCR an upregulation of *amtR *expression upon nitrogen starvation was detectable in different studies [7,8,11-13], although an AmtR consensus site was identified upstream of the *amtR *gene [5]. The reason for the lack of autoregulation was unclear until now, however, compared to other AmtR binding sites the *amtR *upstream sequence differs in two points (i) the presence of a G instead of T at position 2 of the core motif and (ii) in the number of spacing nucleotides between the two half sites, 3 instead of 4. To investigate the importance of these differences, gel shift experiments were carried out using AmtR-MBP, wild type and modified *amtR *promoter regions (Fig. 6). While a G to T nucleotide exchange at position 2 had no significant effect, the introduction of an additional nucleotide clearly led to the binding of AmtR-MBP.

## Discussion

TetR-type regulators are widely distributed among Gram-positive bacteria [9] including corynebacteria. In fact, genome sequence analyses of *Corynebacterium diphtheriae*, *Corynebacterium efficiens*, *C. glutamicum *and *Corynebacterium jeikeium *revealed that these are the most frequently used transcriptional regulators in corynebacteria [13]. Examples for functionally characterized members of the TetR family in *C. glutamicum *besides AmtR are AcnR, CGL2612 and McbR. AcnR binds upstream the *acn *gene encoding aconitase to the putative consensus sequence CAGNACnnncGTACTG, which is highly conserved in corynebacterial and mycobacterial species [14]. CGL2612 is a drug resistance-related regulator with significant structural similarity to the multidrug resistance-related transcription factor QacR from *Staphylococcus aureus *[15]. McbR binds to the consensus motif TAGAC-N6-GTCTA and is involved in the regulation of sulphur metabolism and the synthesis of sulphur-containing amino acids [16,17].

Compared to the other TetR-family members in *C. glutamicum*, AcnR [14] and CGL2612 [15], the AmtR regulon is relatively big and compared to especially McbR [17], the binding consensus is not very strictly conserved. This raises the questions about the similarities and differences in protein-DNA recognition, especially in respect to the well-investigated *E. coli *TetR. For the AmtR protein-DNA interface, mutagenesis revealed an important role for Thr42, Gln53, Ser55, Tyr57, Tyr58, and His59. These residues are either strictly conserved or there are only very conservative replacements observed between AmtR and TetR (Table 1). This finding is consistent with the fact that both repressors recognize the identical "CTAT"-motif in the DNA.

For the remaining three residues of the protein-DNA interface (His43, Arg52, Ala54) differences are observed between AmtR and TetR, which might have implications for binding affinity and specificity. His43 is replaced by arginine in TetR. This arginine forms specific contacts with the purine ring of *G3 *in the TetR-DNA crystal structure [18]. In the model of the AmtR-DNA complex, the distance between His43 and the purine ring is larger due to the shorter histidine side chain, suggesting a weaker interaction (Fig. 5C). Although these differences might also arise from inaccuracies of our model, there are two observations which suggest a real difference between AmtR and TetR. First, a replacement of His43 by alanine leads only to a minor decrease of binding affinity (Fig. 2, 3). Second, the respective G-C base pair does not represent a part of the "CTAT" core motif in AmtR (Fig. 4B) and is non-conserved in AmtR binding sequences [7], while it is conserved in the "CTAT**C**"-motif recognized by TetR. Arg52 of AmtR is replaced by glutamic acid in TetR. The presence of the non-conservative replacement together with the observation that an arginine to alanine replacement has only little effect on AmtR binding affinity suggest that the Arg52 side chain does not play a major role for the DNA interaction.

Another difference is observed at position 54: The respective alanine of AmtR is equivalent to Pro39 of TetR. Ala 54 forms interactions with the methyl group of *T-6*. These interactions, however, seem only to play a minor role for the affinity and specificity of DNA recognition. This is evidenced by the observation that a replacement by glycine has only small effects on binding affinity. In addition, the contact to the methyl-group of base *T-6 *seems not to mediate a specific recognition since a replacement of thymidine by guanosine still allows an interaction (Fig. 5D, Fig. 6). In contrast, Pro39 of TetR contacts two thymidines of adjacent base pairs (*T5 *and *T-6 *according to the *amtR *nomenclature; Fig. 4B) and a replacement by glutamate was shown to have a significant effect on binding affinity and specificity [19]. These findings suggest that Ala54 of AmtR has a smaller role than Pro39 in TetR for promoter recognition.

In summary, at least the differences at sequence positions 43 and 54 might explain the larger sequence variability of DNA recognition by AmtR compared to TetR. Although AmtR tolerates a certain amount of variability within the DNA half-sites, their correct spacing appears to be crucial for binding. The "CTAT" half-sites are separated by a five-residue spacer in TetR, but by six residues in AmtR. As demonstrated by the experiment in Fig. 6, this spacing is essential for the AmtR-DNA interaction. Due to the differences of the spacer length between TetR and AmtR, the structural consequences of this feature on the orientation of the binding heads or the curvature of the DNA cannot be deduced from the present model, but have to await the elucidation of a crystal structure of AmtR in complex with DNA. Future work will now concentrate on the crystallization of AmtR without and together with bound DNA. Using DNA fragments with alterations of the consensus motif and the kind and number of spacing nucleotides, this approach will allow to study functional and structural flexibility of DNA binding domains. Furthermore, the co-crystallization of AmtR with signal transduction protein GlnK will provide the first structural information about an asymmetric GlnK interaction complex. Today, besides the interaction with AmtR, which most likely acts as a dimer [5], exclusively interactions with threefold symmetry were reported, e.g. of the trimeric GlnK complex with trimeric ammonium transporter AmtB, or with the hexameric key enzyme for arginine biosynthesis NAGK [20].

## Conclusion

Molecular biology and biochemistry approaches such as site-directed mutagenesis, gel retardation assays and SPR are, especially in combination with molecular modelling, powerful tools to identify crucial amino acids for DNA binding. In this study, we could show that besides by distinct amino acids of the TetR family protein AmtR, DNA binding is influenced by nucleotides not only of the conserved binding motif but also by spacing nucleotides in *C. glutamicum*.

## Methods

### Bacterial strains and growth

*C. glutamicum *wild type ATCC 13032 [21] was grown at 30°C in MOPS-buffered minimal medium with glucose as carbon source as described [22].

### General molecular biology techniques

For plasmid isolation, transformation, and cloning standard techniques were used [23]. *E. coli *strain DH5α *mcr *[24] was used as cloning host.

### Site directed mutagenesis

For expression and purification of AmtR variants, point mutations were inserted into *amtR *via two-step-PCR using the oligonucleotides amtR-PstI-rev, amtRw/oATG-BamHI-fw and mutagenesis primers (Table 2). For amplification *C. glutamicum *ATCC13032 cell were used as a template. In the first step the mutagenesis primer was used as a forward primer, with amtR-PstI-rev as reverse primer. After denaturation at 96°C for 30 sec the primers were aligned at 65°C for 30 sec and elongation was performed at 72°C for 30 sec. The cycle was repeated 30 times. The resulting fragment served as a reverse primer in the second step, with amtRw/oATG-BamHI-fw as forward primer. Here 58°C was used as annealing temperature and elongation was carried out for 45 sec. The resulting fragment was cloned into the vector pMalc2 via the restriction sites *Bam*HI and *Pst*I. All plasmids constructed (Table 3) were sequenced for control.

**Table 2:** Oligonucleotides used in this study.

**Designation**	**Sequence (5' → 3')**	**Application**
amtRw/oATG-BamHI-fw	GGTCGGATCCGCAGGAGCAGTGGG	Cloning of *amtR *into pMalc2

amtR-PstI-rev	GGCGCCTGCAGTTATTTCGCGTCAGCCTGC	Cloning of *amtR *into pMalc2

amtR23-fwd	TCCTCGCGAGGCGATTCTTGACG	Mutagenesis of *amtR*

amtR25-fwd	CGAGGAGATTGCTGACGCCTCTG	Mutagenesis of *amtR*

amtR30-fwd	CGCCTCTGCTGCGCTTTTCACCC	Mutagenesis of *amtR*

amtR31-fwd	CTCTGCTGAGGCTTTCACCCGTC	Mutagenesis of *amtR*

amtR32-fwd	TGCTGAGCTTGCCACCCGTCAAG	Mutagenesis of *amtR*

amtR33-fwd	TGAGCTTTTCGCCCGTCAAGGCT	Mutagenesis of *amtR*

amtR36-fwd	CACCCGTCAAGCCTTCGCAACAA	Mutagenesis of *amtR*

amtR40-fwd	CTTCGCAACAGCCTCCACGCATC	Mutagenesis of *amtR*

amtR42-fwd	AACAACCTCCGCGCATCAAATCG	Mutagenesis of *amtR*

amtRH43A	CAACCTCCACGGCTCAAATCGCTG	Mutagenesis of *amtR*

amtR50-fwd	TGATGCCGTGGCAATCCGCCAAG	Mutagenesis of *amtR*

amtR51-fwd	TGCCGTGGGAGCCCGCCAAGCCT	Mutagenesis of *amtR*

amtR52-fwd	CGTGGGAATCGCCCAAGCCTCGC	Mutagenesis of *amtR*

amtR53-fwd	GGGAATCCGCGCAGCCTCGCTCT	Mutagenesis of *amtR*

amtRA54A	GAATCCGCCAAGGCTCGCTGTATTATC	Mutagenesis of *amtR*

amtR55-fwd	CCGCCAAGCCGCGCTGTATTATC	Mutagenesis of *amtR*

amtR56-fwd	CCAAGCCTCGGCGTATTATCACT	Mutagenesis of *amtR*

amtR57-fwd	AGCCTCGCTGGCTTATCACTTCC	Mutagenesis of *amtR*

amtR58-fwd	CTCGCTGTATGCTCACTTCCCGT	Mutagenesis of *amtR*

amtR59-fwd	GCTGTATTATGCCTTCCCGTCCA	Mutagenesis of *amtR*

amtR63-fwd	CTTCCCGTCCGCGACGGAAATCT	Mutagenesis of *amtR*

amtR70-fwd	CTTCCTCACCGCGCTGAAATCTA	Mutagenesis of *amtR*

probe-amtB-fw	GCT GGG CTA GAA ACC CGA	*amtB *promoter fragment for gel retardation assays (nt position -298 to -1)

probe-amtB-rev	GCG TGG ATG ACC TCC TTT G	*amtB *promoter fragment for gel retardation assays (nt position -298 to -1)

binding1_amtB-fw	TAAATTACCTGTTAAACTATAGAAAATATC	*amtB *promoter fragment for SPR (nt position -186 to -156)

bind1_amtB-rew-2	GATATTTTCTATAGTTTAACAGGTAATTTA	*amtB *promoter fragment for SPR (nt position -186 to -156)

amtRbs-f	GCCCGTGGTGTGCTCACCAATG	*amtR *promoter fragment for gel retardation assays (nt position -291 to -63)

amtRbs-r	CAGAGTTCCTATTTGGTATCGATTTCACGGGC	*amtR *promoter fragment for gel retardation assays (nt position -291 to -63)

amtRbsG-T-r	CAGAGTTCCTATTTGGTATAGATTTCACGGGC	*amtR *promoter fragment for gel retardation assays

amtRbs+N-r	CAGAGTTCCTATTATGGTATCGATTTCACGGGC	*amtR *promoter fragment for gel retardation assays

**Table 3:** Plasmids used in this study

**Plasmid**	**Genotype/Description**	**Reference**
pMalc2	*ptac*, Ap^R^, *ori *ColE1, *malE*, *lacZα*, *lacI^*q*^, E. coli-*Vektor for protein purification	NEB, Schwalbach

pMalc2amtR	pMalc2, p_tac_-*malE-amtR*	This work

pMalc2amtR *Glu23Ala	pMalc2amtR, point mutation in *amtR *for AmtR*Glu23Ala variant	This work

pMalc2amtR* Arg52Ala	pMalc2amtR, point mutation in *amtR *for AmtR*Arg52Ala variant	This work

pMalc2amtR* Gly50Ala	pMalc2amtR, point mutation in *amtR *for AmtR*Gly50Ala variant	This work

pMalc2amtR* Ile51Ala	pMalc2amtR, point mutation in *amtR *for AmtR*Ile51Ala variant	This work

pMalc2amtR* Thr40Ala	pMalc2amtR, point mutation in *amtR *for AmtR*Thr40Ala variant	This work

pMalc2amtR* Thr42Ala	pMalc2amtR, point mutation in *amtR *for AmtR*Thr42Ala variant	This work

pMalc2amtR*Glu30Ala	pMalc2amtR, point mutation in *amtR *for AmtR*Glu30Ala variant	This work

pMalc2amtR*Gly36Ala	pMalc2amtR, point mutation in *amtR *for AmtR*Gly36Ala variant	This work

pMalc2amtR*Leu31Ala	pMalc2amtR, point mutation in *amtR *for AmtR*Leu31Ala variant	This work

pMalc2amtR*Phe32Ala	pMalc2amtR, point mutation in *amtR *for AmtR*Phe32Ala variant	This work

pMalc2amtR*Thr33Ala	pMalc2amtR, point mutation in *amtR *for AmtR*Thr33Ala variant	This work

pMalc2amtRAla54Gly	pMalc2amtR, point mutation in *amtR *for AmtR*Ala54Gly variant	This work

pMalc2amtRHis43Ala	pMalc2amtR, point mutation in *amtR *for AmtR*His43Ala variant	This work

pMalc2amtR*Gly36Ala	pMalc2amtR, point mutation in *amtR *for AmtR*Gly36Ala variant	This work

pMalc2amtR*Thr40Ala	pMalc2amtR, point mutation in *amtR *for AmtR*Thr40Ala variant	This work

pMalc2amtR*Thr42Ala	pMalc2amtR, point mutation in *amtR *for AmtR*Thr42Ala variant	This work

pMalc2amtR*Gly50Ala	pMalc2amtR, point mutation in *amtR *for AmtR*Gly50Ala variant	This work

pMalc2amtR*Ile51Ala	pMalc2amtR, point mutation in *amtR *for AmtR*Ile51Ala variant	This work

pMalc2amtR*Arg52Ala	pMalc2amtR, point mutation in *amtR *for AmtR*Arg52Ala variant	This work

pMalc2amtR*Gln53Ala	pMalc2amtR, point mutation in *amtR *for AmtR*Glu53Ala variant	This work

pMalc2amtR*Ser55Ala	pMalc2amtR, point mutation in *amtR *for AmtR*Ser55Ala variant	This work

pMalc2amtR*Leu56Ala	pMalc2amtR, point mutation in *amtR *for AmtR*Leu56Ala variant	This work

pMalc2amtR*Tyr57Ala	pMalc2amtR, point mutation in *amtR *for AmtR*Tyr57Ala variant	This work

pMalc2amtR*Tyr58Ala	pMalc2amtR, point mutation in *amtR *for AmtR*Tyr58Ala variant	This work

pMalc2amtR*His59Ala	pMalc2amtR, point mutation in *amtR *for AmtR*His59Ala variant	This work

pMalc2amtR*Leu70Ala	pMalc2amtR, point mutation in *amtR *for AmtR*Leu70Ala variant	This work

pMalc2amtR*Leu71Ala	pMalc2amtR, point mutation in *amtR *for AmtR*Leu71Ala variant	This work

### Protein purification

*E. coli *BL21 [25] freshly transformed with pMalc2amtR* vectors was used for inoculation of 300 ml LB containing 2% glucose. Bacteria were grown over night at 37°C and used to inoculate 800 ml fresh LB containing 2% glucose at an OD_600 _of 0.1. The culture was grown to an OD_600 _of 0.5 and subsequently induced with 0.3 mM IPTG. After 4 h of incubation the cells were harvested (3,000 × g, 10 min, 4°C) and the pellet was resuspended in 25 ml purification buffer (20 mM Tris-HCl, pH 7.4, 200 mM NaCl, 1 mM EDTA). The solution was sonicated three times for 30 sec at 70% (Bandelin Sonoplus UW2070, Berlin) and centrifugated for 10 min with 14,000 × g at 4°C. The supernatant was loaded onto a 1 ml MBP-Trap column (GE Healthcare, Munich), washed with 10 column volumes purification buffer and protein bound was eluted with 20 mM maltose in purification buffer.

### Gel retardation experiments

Target DNA for gel shift assays was synthesized by PCR (for the primers used, see Table 2) and was purified by agarose gel electrophoresis. To label the DNA and to prepare the reaction mixture for the gel shift assay, the DIG gel shift kit (Roche, Mannheim) was used as recommended by the supplier. Separation by gel electrophoresis was performed in native 6% polyacrylamide gels (Anamed Electrophorese GmbH, Darmstadt, Germany) using 0.5 × Tris-borate-EDTA buffer as the running buffer. Subsequently, the labelled DNA was transferred to a nylon membrane (Roche, Mannheim, Germany) by electro blotting as described in the protocol of the DIG gel shift kit (Roche, Mannheim, Germany). For detection of the labelled DNA, X-ray film was used.

### Preparation of amtB_p _DNA for SPR

Thirty nucleotide synthetic oligonucleotides containing *amtB*_p _(forward: 5'TAAATTA CCTGTTAAACTATGAAAATATC; backward: 5'-GATATTTTCTATAGTTTAACAGG TAATTTA-3') or a nonspecific DNA sequence (5'CGCGATAATCTTTGCTAACCCTTT TGAGTT-3'; backward: 5'-AACTCAAAAGGGTTAGCAAAGATTATCGCG-3') were hybridized and used for analyses without further purification. The forward 30-nt oligonucleotides carried a biotin at the 3'-end. All oligonucleotides were purchased with or without modification from MWG Biotech (Ebersberg, Germany). The concentration of the hybridized DNA was determined using a pEQlab (Erlangen) Nanodrop Spectrophotometer.

### Surface plasmon resonance (SPR)

SPR measurements with AmtR from *C. glutamicum*, overexpressed as maltose binding protein fusion in *E. coli *BL21, were performed using a BIAcoreX instrument operated at 25°C (Biacore, Uppsala, Sweden). For interaction analyses of an *amtB *promoter fragment with AmtR and AmtR variants 3'end biotinylated DNA comprising *amtB*_p _DNA sequence or a non-specific DNA sequence was immobilized on the surface of Biacore CM5 chips. For this purpose, the chip surface was activated with 35 μl of a mixture of 50 mM N-hydroxysuccinimide and 20 mM N-ethyl-N-(3-dimethylaminopropyl)-carbodiimide-hydrochloride (Biacore, Uppsala, Schweden). After coupling of 3,000 RU neutravidine (5 μM in 10 mM Na-acetate, pH 5.0), to the chip, 35 μl 1 M ethanolamine (Biacore, Uppsala, Sweden) was used to inactivate the remaining reactive carboxyl groups on the chip. Hybridized non-specific DNA was coupled in flow cell 1 and *amtB*_p _DNA in flow cell 2. In all measurements HBS-EP was used as a running buffer. The flow rate was 5 μl/min during coupling and 40 μl/min for all measurements. To regenerate the chip surface the dissociation of the *amtB*_P_-AmtR or amtB_P_-AmtR* variants complex was stopped by injection of 80 μl HBS-EP buffer at 40 μl/min after each injection. AmtR*-MBP variant concentrations of 2170 nM, 10870 nM, 28260 nM and 43470 nM were used. Additionally 43 nM and 870 nM of AmtR were used for the SPR measurements. Evaluation of the data was performed using BiaEvaluation 4.0 Software (Biacore, Uppsala, Schweden). The titrations for the kinetic measurements have been carried out twice for each AmtR variant.

### Model building

The structure of AmtR from *C. glutamicum *in complex with DNA was modeled based on the crystal structure of the TetR repressor/operator complex from *E. coli *(PDB code: 1QPI) [18]. Both proteins share a sequence identity of 37% in the DNA binding domain, which is also reflected in the significant E-value of 10^-5 ^for the respective sequence alignment. Since the remaining parts of the two repressors are highly divergent in sequence, modeling was restricted to the DNA binding domain. The two DNA binding heads of dimeric AmtR were modeled separately using Swiss-Model [26] and the structure of the protein DNA complex was obtained by assuming an identical interface geometry as in the TetR-DNA complex.

The model was subsequently refined by 100 steps of energy minimization using the Sybyl 7.3 program package (Tripos Inc.). The quality of the structure was assessed using Procheck [27] and Whatcheck [28] and did not reveal any steric clashes or unfavorable geometries thus confirming the overall good quality of the model. Finally, contacts between the AmtR and its target DNA were retrieved using LIGPLOT [29]. Visualization and analysis of the model features were carried out using the program Discovery Studio Visualizer (Accelrys Software Inc.).

## Authors' contributions

DM, NJ and KH carried out cloning, site-directed mutagenesis experiments, protein purifications, gel retardation assays and SPR. Molecular modelling was carried out CJ. HS and AB conceived the study and participated in its design and coordination and drafted the manuscript. All authors read and approved the final manuscript.
